# Health systems strengthening to optimise scale-up in global mental health in low- and middle-income countries: lessons from the frontlines. A re-appraisal

**DOI:** 10.1017/S2045796020000475

**Published:** 2020-06-15

**Authors:** I. Petersen, A. van Rensburg, S. G. Gigaba, Z. B. P. Luvuno, L. R. Fairall

**Affiliations:** 1 Centre for Rural Health, University of KwaZulu-Natal, Durban, South Africa; 2 Knowledge Translation Unit, University of Cape Town, Cape Town, South Africa; 3 King's Global Health Institute, King's College London, London, UK

**Keywords:** Chronic care, collaborative care, common mental disorders, health service research

## Abstract

Against the backdrop of mounting calls for the global scaling-up of mental health services – including quality care and prevention services – there is very little guidance internationally on strategies for scaling-up such services. Drawing on lessons from scale-up attempts in six low- and middle-income countries, and using exemplars from the front-lines in South Africa, we illustrate how health reforms towards people-centred chronic disease management provide enabling policy window opportunities for embedding mental health scale-up strategies into these reforms. Rather than going down the oft-trodden road of vertical funding for scale-up of mental health services, we suggest using the policy window that stresses global policy shifts towards strengthening of comprehensive integrated primary health care systems that are responsive to multimorbid chronic conditions. This is indeed a substantial opportunity to firmly locate mental health within these horizontal health systems strengthening funding agendas. Although this approach will promote systems more enabling of scaling-up of mental health services, implications for donor funders and researchers alike is the need for increased time commitments, resources and investment in local control.

To reduce the ever-increasing burden of mental health problems in resource-scarce contexts, the recent Lancet Commission on global mental health and sustainable development has identified the need for, *inter alia*, scale-up of access to quality care as well as prevention efforts. Key innovations/implementation strategies recommended to promote rapid scale-up include task sharing; care that is balanced across levels and different sectoral delivery platforms; the use of digital technologies and interventions to enhance demand for care (Patel *et al*., 2018). However, although scale-up is a clear imperative as the next phase for Global Mental Health, exactly how this needs to unfold is less clear. Although guidance has been generated for researchers and implementers for other vertical health programmes – specifically for HIV and substance abuse prevention – similar strategies are lacking for mental health intervention scale-up across different contexts (Power *et al*., 2019). Grounded in lessons emerging from efforts that use some of these key innovations to scale-up integrated mental health care at the district level in low- and middle-income countries (LMICs), we suggest the need to locate these strategies within a broader enabling health system strengthening agenda so as to optimise institutionalisation and sustainability of scale-up efforts.

We show below firstly, how the lessons gleaned from the Emerging Mental Health Systems in Low-and Middle-Income Countries (Emerald) research consortium (Semrau *et al*., 2015) suggest that health systems reforms to support chronic care provide an enabling framework for integrated mental health care. The need for a people-centred approach to these systems reforms is also underlined by findings from this study. The latter emphasises the need for health care provisioning to be responsive to people's needs and expectations, placing at the epicentre, empowerment and engagement of individuals, families and communities, to promote and protect their health and that of their communities as participants and beneficiaries (Sheikh *et al*., 2014). A people-centred approach also speaks to the Lancet Commission's emphasis on engagement of people in mental health care provisioning and increased emphasis on prevention efforts across the life-course. Secondly, we provide exemplars from the frontlines of reforms to develop people-centred health systems that are enabling of multimorbid chronic disease management in South Africa; and which provide policy window opportunities for embedding mental health scale-up strategies into these reforms. Lastly, we identify the implications of lessons learned from these efforts for the global mental health scale-up agenda.

## Lessons from the Emerald cross-country study

A key output of the Emerald study was lessons from the scale-up attempts of evidence-based integrated mental health care packages in six LMICs. A noteworthy finding was that an add-on training approach, which has characterised much of the implementation of the WHO Mental Health Gap Action Programme (mhGAP), was insufficient to sustain integration, with the need for system strengthening interventions to enable integration emphasised (Petersen *et al*., 2019*b*). More specifically, this study reinforced the need for strengthening of the basic building blocks of the health care system for chronic care to provide an enabling and supportive system and environment for integrated mental health care. Systems reforms to support chronic care are necessary to respond to the global changing disease burden from predominantly acute conditions to chronic ones, that often include comorbid mental health conditions. Based on a review of available evidence, Thornicroft *et al*. suggest that the World Health Organization's Innovative Care for Chronic Care Framework (ICCCF) provides a useful framework to this end (Thornicroft *et al*., 2019). The ICCCF identifies three levels of the health system in a socioecological fashion: the policy and population level, meso or district/sub-district level, as well as the family and community levels. At the policy and population levels, policies and regulations that are health-promoting, for example, regulations relating to alcohol consumption and which may need to be implemented by other sectors, are important, as are governance issues, particularly of an inter-sectoral nature (Petersen *et al*., 2019*b*). At the meso-level, governance remains important, with frontline managers being responsible for translating and adapting programmes within organisational and systems governance architecture to customise and embed them within the system (Scott *et al*., 2014). To this end, continuous quality improvement (CQI) was identified as an important vehicle for embedding implementation strategies in the Emerald study (Petersen *et al*., 2019*b*). In relation to human resources, person-centred care, which is subsumed under people-centred care, is central to care provision at the meso-level. It focuses on the totality of the person from a holistic biopsychosocial perspective; with clinical communication skills a helpful tool for understanding a person holistically (Stuart *et al*., 2000). This includes the identification of common mental disorders (CMDs) that may accompany patients' presenting physical complaints in PHC settings, as found in the Emerald study (Petersen *et al*., 2019*b*). Additionally, at the meso-level, clinical decision support tools are highlighted as helping to support providers with clinical expertise and skills to deliver the best possible care according to the latest evidence. In the Emerald study, integrated chronic care guidelines that included mental health were found helpful to embed task-sharing of mental health within the tasks of primary care providers (Petersen *et al*., 2019*b*). Furthermore, integrated clinical information systems that are central to capture information on multiple conditions, so as to optimise multimorbidity care as well as facilitate continuity of care necessary for long-term conditions, are important; as is delivery system re-design to promote collaborative team-based care which has been found to be effective for PHC mental health-care integration in high-income countries (Archer *et al*., 2012). Being able to leverage existing established referral pathways and collaborations between levels of care and different sectors necessary for balanced care; as well as systems for dispensing chronic medication and information systems for tracing *lost to care* patients so as to reduce non-adherence and the chance of relapse were all found helpful for facilitating uptake and embedding of the mental health integration strategies in the Emerald study (Petersen *et al*., 2019*b*). At the micro-level, achieving activated empowered and informed people, who have control over their health and living with their condition over the long-term, emerged as an important issue in the Emerald study, and is central and in step with a people-centred approach. Micro-level interventions, however, require a robust community sub-system that includes community health workers, who interface with the formal health system, as well as households, community structures such as faith-based organisations, civic groups and local political structures, and other sectors providing services at a community level (Petersen *et al*., 2019*b*). Establishing community supports such as peer-led support groups as well as mechanisms for tracing and tracking patient progress is emphasised. Promotion and prevention interventions are also key at this level, with people having control of their health and that of their communities ultimately being the hallmark of people-centredness. The Emerald study found existing community health worker outreach teams provided an important platform that could be leveraged for task sharing of psychosocial interventions and psychoeducation to improve demand for services as well as for tracing of non-adherent patients (Petersen *et al*., 2019*b*).

## Exemplar of optimising health system reform policy windows: the case of South Africa

**Fig. 1.**
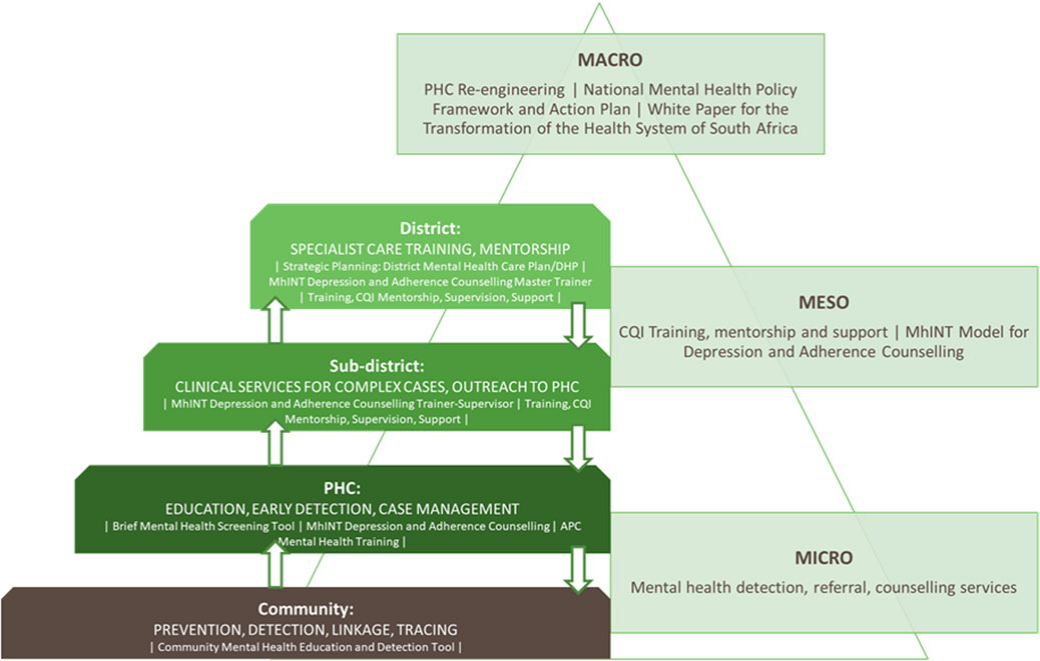
Integration of mental health into existing platforms at meso and micro levels in the KwaZulu-Natal province, South Africa.

Recent calls for health reforms in LMICs towards horizontal chronic people-centred health systems (Agyepong *et al*., 2018; Thornicroft *et al*., 2019) provide a policy window to embed mental health into routine comprehensive primary health care so as to become part of reformed service delivery models from the outset rather than being seen as add-ons (Kingdon, 2010). We demonstrate how such health reforms in South Africa (Pillay and Baron, 2011; Mahomed and Asmall, 2015), from vertical to horizontal services, have provided opportunities to integrate mental health into routine care. An integrated collaborative care package for CMDs originally developed and tested through the Programme for Improving Mental Health Care (PRIME) research collaboration (Petersen *et al*., 2019*a*), was re-packaged for scale-up by the Mental health Integration Programme (MhINT) and currently being evaluated through the Southern African Mental health InTegration (SMhINT) project (https://www.crh.ukzn.ac.za). The implementation and evaluation of this scale-up package is being undertaken in one initial pilot district in the KwaZulu-Natal (KZN) province. This is being executed through a collaboration between MhINT technical support staff, SMhINT researchers and provincial and district Department of Health managers. In line with recommendations from Emerald, CQI is being used as the overall methodological framework for identifying what works, for whom and under what conditions so as to inform how the package may need to be strengthened prior to further scale-up. Whilst the collaborative care package was developed for the PHC platform, it is emphasised that mental health integration is a system-wide endeavour. As such the MhINT programme is comprised of several interventions targeting different levels of the health care system (macro, meso and micro) to ensure an enabling environment for integration along the treatment cascade. This is depicted in Fig. 1, which was developed in collaboration with the KZN provincial Mental Health and Substance Abuse Directorate.

At the Provincial level, the MhINT programme has engaged with the Mental Health and Substance Abuse Directorate and the Directorate for District Health Services to ensure that the testing of the implementation of the MhINT package is aligned with the existing resources and re-organisation of services according to the current health policy reforms. Lessons generated from CQI processes at the meso- and micro-levels have uncovered, *inter alia*: (i) the need for a review of the mental health data elements in the district health information system to facilitate improved monitoring of mental health services along the cascade of care in PHC; (ii) validated mental health screening instruments for use in PHC facilities and at the community level and (iii) the development of standardised operating procedures for administration of these tools.

At the meso level, and working within the initial scale-up district, the MhINT programme has to be continuously responsive to the unique contextual factors that support or hinder integration of mental health care. To overcome the challenge of the integration of mental health into PHC being seen as additional work to existing responsibilities; MhINT aligns its activities with the Integrated Clinical Services Management (ICSM) model of managed care for chronic conditions, as well as the South African Ideal Clinic guidelines. This is being done through strengthening the existing PHC process of clinical management such as (i) strengthening mental health screening by providing a validated screening tool to clinicians tasked with proving screening services (Bhana *et al*., 2019); (ii) enhancing clinicians' training on the existing clinical decision-making tools for managing chronic diseases [integrated chronic care guidelines called Adult Primary Care (APC)] (Fairall *et al*., 2015), so that these guides can be used to diagnose and manage CMDs and (iii) strengthening referral pathways by training identified PHC-based staff to provide a structured psychosocial counselling intervention to ensure a mental health service within PHC for CMDs. Furthermore, MhINT provides a mentorship and supervision model to different cadres at the different levels of the health care system to foster the spirit of collaboration and strengthen the existing referral pathways. For instance, in the initial scale-up district, district psychologists have been equipped with skills to train and support mid-level mental health care workers in the system, who within the model, would be expected to support and supervise the PHC-based staff tasked with providing the psychosocial intervention at the PHC level to patients with CMDs (which to date have included HIV counsellors and enrolled nurses). Additionally, PHC supervisors in this initial site were also oriented to CQI, with a MhINT CQI mentor providing support during the early stages of implementation.

At the micro level, the MhINT programme provides further support for the integration of mental health into routine outreach team activities through (i) the development and evaluation of a Community Mental Health Psychoeducation and Detection (CMED) tool for use by community outreach team members to enhance community mental health literacy, as well as detect community members who may require mental health services; and (ii) contributing to the development of an accompanying training curriculum for these outreach teams led by the South Africa HIV Addiction Technology Transfer Centre (ATTC) network.

Importantly, the structures and relationships fostered by MhINT have also been leveraged to strengthen the health system for tuberculosis (TB) patients who are particularly vulnerable to CMDs. Towards this end, a work package of the National Institute for Health Research (NIHR) Global Health Research Unit on Health System Strengthening in Sub-Saharan Africa (ASSET) (NIHR, 2019) for strengthening people-centred TB care is presently being layered into the health system in the same district site as the MhINT programme. Taking an embedded participatory approach, this work package aims to develop person-centred TB care that can provide services and support to people with TB according to their individual, family and community needs, ultimately to reduce a TB mortality rate that is almost double that of the national average. By adapting critical TB programme dimensions such as screening and diagnosis to be less judgemental and stigmatising, while also considering the complexities of multimorbidities (especially with depression and HIV), this ASSET work package is generating bottom-up solutions to strengthen the district health system for improved people-centred care of people burdened by mental–physical multimorbidities. In a practical sense, this requires constant iterative cycles of planning and implementation, driven by a collaborative team consisting of researchers and government and community stakeholders, as well as programmatic flexibility to allow for action-oriented intervention development, testing and evaluation.

## Implications for the global mental health scale-up agenda

These lessons from the ground have implications for the way in which we, as the global mental health community, approach scaling-up of increased access to mental health care in scarce-resource contexts.

In the first instance, they sound a cautionary note to the call for global mental health financing similar to that invested in eradicating HIV and other epidemics (Kleinman *et al*., 2016; Vigo *et al*., 2019). Such global health initiatives in the past – especially in African countries – have operated vertically, bypassed country systems, influenced policy to suit multilateral agendas and have not sufficiently harmonised with other local initiatives (Mwisongo and Nabyonga-Orem, 2016); resulting in untended consequences on health systems in the recipient countries. For example, HIV donor funding has been found to ‘crowd out’ other health issues – especially in contexts where there are limited human resources for health and where providers' attention becomes focused solely on the targets and indicators of the vertical donor funded priority programme (Grepin, 2012). This has often been to the detriment of other programmes and sustainability. This approach in the global mental health sphere, may thus unintentionally entrench the perception of mental health as a vertical programme, and work against the goal of comprehensive integrated care and sustainability (Yasamy *et al*., 2011). In light of the increasing global burden of multimorbid chronic conditions, we suggest an expansion of the global mental health scale-up funding agenda to ensure that mental health is firmly part of funding streams for health systems strengthening to adequately prevent and manage multiple co-existing chronic conditions. By investing both in systems as enabling environments as well as in horizontal programming, incremental changes are more likely to be sustainable and ultimately, cost-effective.

Second, the inclusion of dialogical action principles such as the adoption of co-learning and co-design between researchers and communities are important to foster acceptability and sustainability. This requires a recasting of partnerships and collaborations between research institutes, organisations and funding bodies situated in high-income geographies, and researchers, policy-makers and implementers at a local level in LMICs, from expert-driven to emphasising co-learning, support and respect (Horner, 2019); and balancing universalisation and standardisation on the one hand, with geographic and cultural variation on the other (Horner, 2019). This may require increased time commitments, resources and investment in local control; with the flipside being that researchers and funders have less control over the ultimate outcomes of the intervention (Beran *et al*., 2018).

Lastly, global policy shifts, as well as emerging policy shifts in LMICs towards strengthening of comprehensive integrated primary health care systems that are responsive to multimorbid chronic conditions, provide a policy window for the global mental health community to ensure that mental health is firmly integrated within these multimorbidity policy shifts and accompanying implementation strategies from the outset of such reforms. This should assist in addressing the low priority afforded to mental health in LMICs historically, alleviate the problem of it being seen as an add-on to the existing services, improve health outcomes, reduce costs as well as potentially reduce institutional stigma (Thornicroft *et al*., 2019). It is also aligned with the recent call for a *Lancet* editorial calling for the global mental health community to ‘transform global health into a movement with mental health’ (Horton, 2019).

## Note

This editorial replaces the following retracted editorial: Petersen I, van Rensburg A, Gigaba S, Luvuno ZPB, Fairall L: Health systems strengthening to optimise scale-up in global mental health in low- and middle-income countries: lessons from the frontlines. *Epidemiology and Psychiatric Sciences* 2020, 29:e101. This previous editorial was retracted at the request of the authors on the basis of discussions with the KwaZulu-Natal (KZN) Department of Health, where it came to light that some of the information on MhINT that was divulged in the previous editorial had overstated the scope that as covered through MhINT for the KZN Department of Health – and should thus not have been included. This new editorial has updated the section on MhINT of this retracted editorial so that this section is a more accurate reflection of the scope of the MhINT programme for the KZN Department of Health. There have been no changes to any other aspects of the previous editorial.
